# Supplementary material for: Capacity for compensatory cyclin D2 response confers trametinib resistance in canine mucosal melanoma
Source: bioRxiv. 2025 Apr 26:2025.04.24.650512. Preprint. [Version 1] doi: 10.1101/2025.04.24.650512 (PMC12190752; doi:10.1101/2025.04.24.650512)
Supplement: 1 [file NIHPP2025.04.24.650512V1-supplement-1.pdf]

bioRxiv preprint doi: https://doi.org/10.1101/2025.04.24.650512; this version posted April 26, 2025. The copyright holder for this preprint (which was not certified by peer review) is the author/funder. This article is a US Government work. It is not subject to copyright under 17 USC 105 and is also made available for use under a CC0 license.

# Supplemental Figure S1

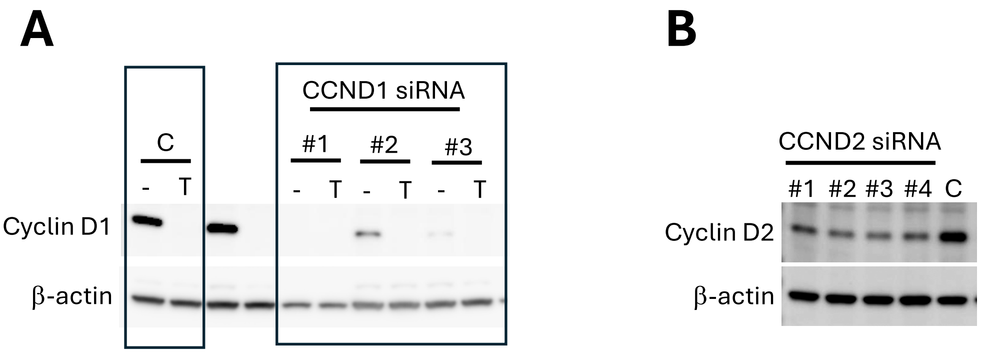

bioRxiv preprint doi: https://doi.org/10.1101/2025.04.24.650512; this version posted April 26, 2025. The copyright holder for this preprint (which was not certified by peer review) is the author/funder. This article is a US Government work. It is not subject to copyright under 17 USC 105 and is also made available for use under a CC0 license.

**Supplemental Figure S1.** siRNA specificity test. A) Three different siRNA constructs targeting canine cyclin D1 gene (CCND1) were tested. M1 cells were transfected with 30 nM siRNA for 24 hours. The cyclin D1 level under trametinib (T) or DMSO control (-) was evaluated. B) Four different siRNA targeting cyclin D2 gene (CCND2) were similarly tested for their specificity. The cyclin D2 protein level was evaluated 24 hours after siRNA transfection.

bioRxiv preprint doi: https://doi.org/10.1101/2025.04.24.650512; this version posted April 26, 2025. The copyright holder for this preprint (which was not certified by peer review) is the author/funder. This article is a US Government work. It is not subject to copyright under 17 USC 105 and is also made available for use under a CC0 license.

# Supplemental Figure S2

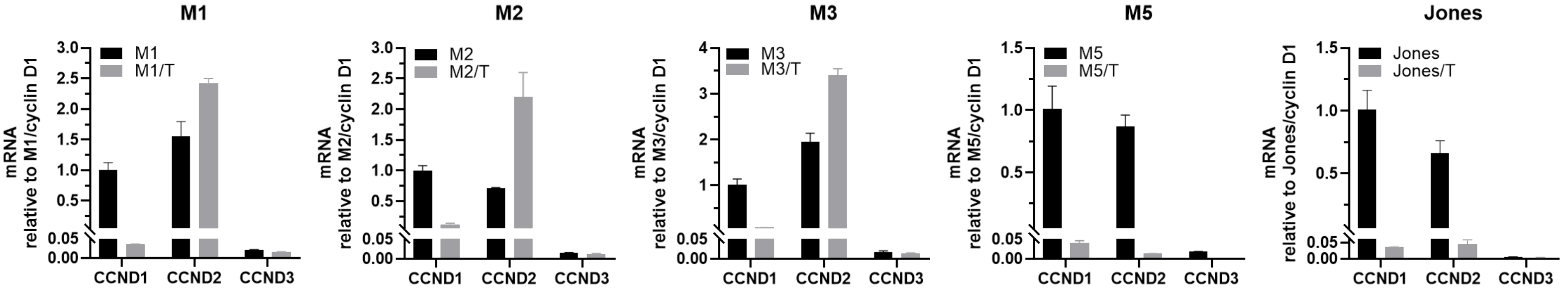

bioRxiv preprint doi: https://doi.org/10.1101/2025.04.24.650512; this version posted April 26, 2025. The copyright holder for this preprint (which was not certified by peer review) is the author/funder. This article is a US Government work. It is not subject to copyright under 17 USC 105 and is also made available for use under a CC0 license.

**Supplemental Figure S2.** Quantitative PCR of CCND1, CCND2, and CCND3. Canine MM cells were treated with 1.0 μM trametinib (T) treatment for 48 hours. The quantity of each mRNA was reference to CCND1 level of control untreated cells in each cell line.

bioRxiv preprint doi: https://doi.org/10.1101/2025.04.24.650512; this version posted April 26, 2025. The copyright holder for this preprint (which was not certified by peer review) is the author/funder. This article is a US Government work. It is not subject to copyright under 17 USC 105 and is also made available for use under a CC0 license.

# Supplemental Figure S3

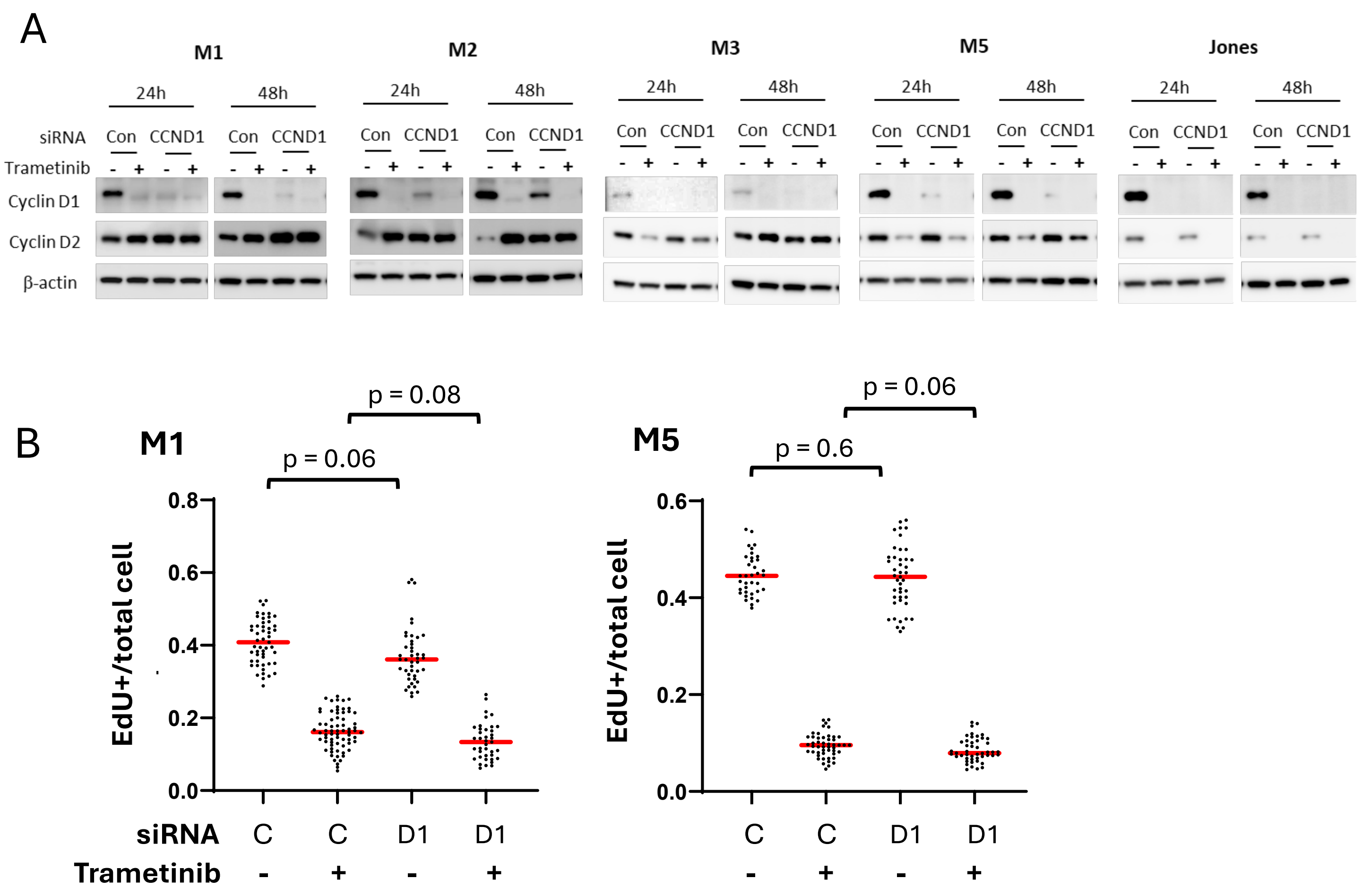

bioRxiv preprint doi: https://doi.org/10.1101/2025.04.24.650512; this version posted April 26, 2025. The copyright holder for this preprint (which was not certified by peer review) is the author/funder. This article is a US Government work. It is not subject to copyright under 17 USC 105 and is also made available for use under a CC0 license.

**Supplemental Figure S3.** Responses of canine MM cells to cyclin D1 knock down (KD). A) Levels of cyclin D1 and D2 in canine MM cells treated with cyclin D1 siNRA followed by trametinib treatment. Cells were transfected with Cyclin D1 (CCND1) or control (Con) siRNA for 24 hours followed by 0.5 μM trametinib treatment for 24 and 48 hours. B) Cyclin D1 knock down (D1) does not significantly affect proliferative capacity in M1 and M5 cells compared to control siRNA (C), regardless of trametinib treatment. Following siRNA transfection for 24 hours, cells were treated with 0.5 μM trametinib (+) or DMSO (-) for 24 hours before EdU labeling. Each dot represents one field of view. The ratios of EdU positive cells and total cell number were used to represent the proliferative activities.

bioRxiv preprint doi: https://doi.org/10.1101/2025.04.24.650512; this version posted April 26, 2025. The copyright holder for this preprint (which was not certified by peer review) is the author/funder. This article is a US Government work. It is not subject to copyright under 17 USC 105 and is also made available for use under a CC0 license.

# Supplemental Figure S4

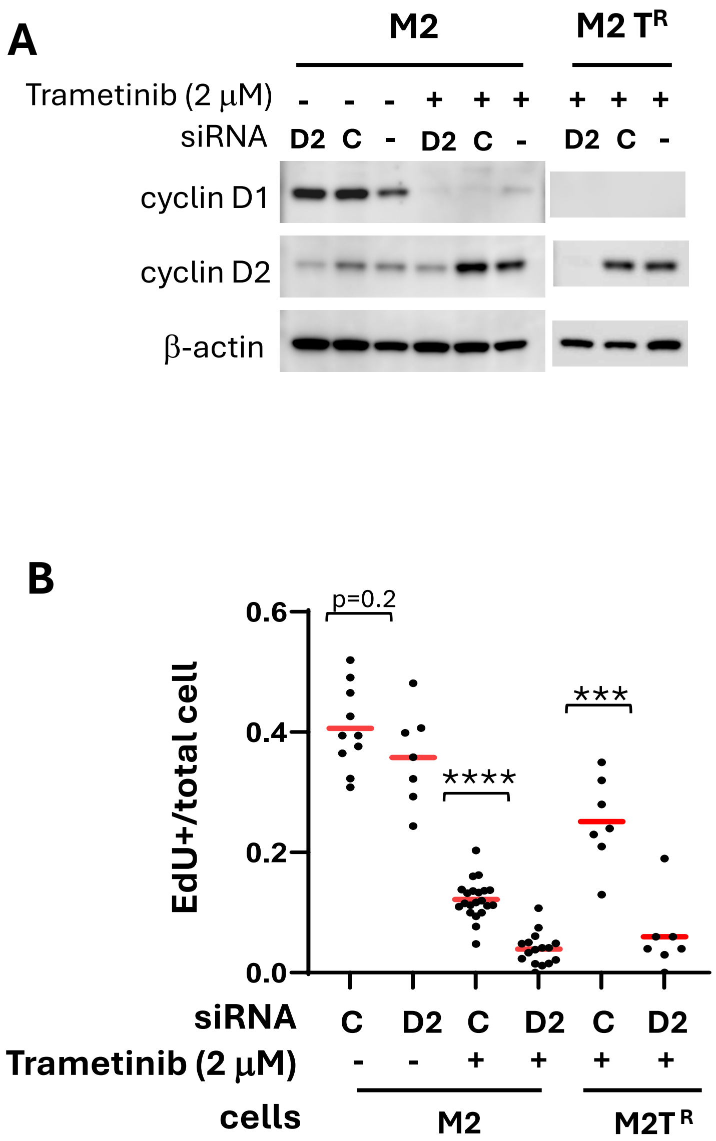

bioRxiv preprint doi: https://doi.org/10.1101/2025.04.24.650512; this version posted April 26, 2025. The copyright holder for this preprint (which was not certified by peer review) is the author/funder. This article is a US Government work. It is not subject to copyright under 17 USC 105 and is also made available for use under a CC0 license.

**Supplemental Figure S4.** Cyclin D2 expression is similarly critical for the proliferative capacity of trametinib-resistant M2 ($M2T^R$) cells. Parental M2 cells were subjected to adaptive conditioned exposure to trametinib to generate $M2T^R$ trametinib resistant cells, as depicted in Figure 4A for $M1T^R$. A) M2 and $M2T^R$ cell were transfected with CCND2 siRNA (D2), control siRNA (C) or no siRNA (-) followed by 24 hours of 2 μM trametinib treatment (2 μM representing the maintenance concentration of trametinib for $M2T^R$ cell culture). CCND2 siRNA diminished cyclin D2 level compared to expression system control (C) or no siRNA (-). B) siRNA KD of cyclin D2 in M2 and $M2T^R$ cells diminished EdU incorporation indicating significantly reduced cell proliferation and increased sensitivity to trametinib.

bioRxiv preprint doi: https://doi.org/10.1101/2025.04.24.650512; this version posted April 26, 2025. The copyright holder for this preprint (which was not certified by peer review) is the author/funder. This article is a US Government work. It is not subject to copyright under 17 USC 105 and is also made available for use under a CC0 license.

# Supplemental Figure S5

**A**

```
A0A8I3Q7A5|CCND2_CNALF MQGRGSGFFLRGRGPRKNRCPEKAGERAGTRLAMELLCCEVDPVRRAVPDANLLYDDRVL   60
    P30279|CCND2_HUMAN ---------------------------------MELLCHEVDPVRRAVRDRNLLRDDRVL   27
                                                        ***** ********* * *** *****

A0A8I3Q7A5|CCND2_CNALF QNLLTIEERYLPQCSYFKCVQKDIQPYMRRMVATWMLEVCEEQKCEEEVFPLAMNYLDRF   120
    P30279|CCND2_HUMAN QNLLTIEERYLPQCSYFKCVQKDIQPYMRRMVATWMLEVCEEQKCEEEVFPLAMNYLDRF   87
                       ************************************************************

A0A8I3Q7A5|CCND2_CNALF LAGVPTPKTHLQLLGAVCMFLASKLKETIPLTAEKLCIYTDNSIKPQELLEWELVVLGKL   180
    P30279|CCND2_HUMAN LAGVPTPKSHLQLLGAVCMFLASKLKETSPLTAEKLCIYTDNSIKPQELLEWELVVLGKL   147
                       ********:******************* *******************************

A0A8I3Q7A5|CCND2_CNALF KWNLAAVTPHDFIEHILRKLPQPSEKLSLIRKHAQTFIALCATDFKFAMYPPSMIATGSV   240
    P30279|CCND2_HUMAN KWNLAAVTPHDFIEHILRKLPQQREKLSLIRKHAQTFIALCATDFKFAMYPPSMIATGSV   207
                       **********************  ************************************

A0A8I3Q7A5|CCND2_CNALF GAAICGLQQDEDVSSLTGDALVDLLAKITNTDVDCLKACQEQIEVVLLNSLQQFRQDQGD   300
    P30279|CCND2_HUMAN GAAICGLQQDEEVSSLTCDALTELLAKITNTDVDCLKACQEQIEAVLLNSLQQYRQDQRD   267
                       ***********:***** ***.:*********************.********:**** *

A0A8I3Q7A5|CCND2_CNALF GSKSEDELDQASTPTDVRDIDL   322
    P30279|CCND2_HUMAN GSKSEDELDQASTPTDVRDIDL   289
                       **********************
```

**B**

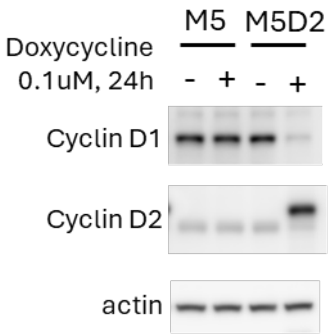


**C**

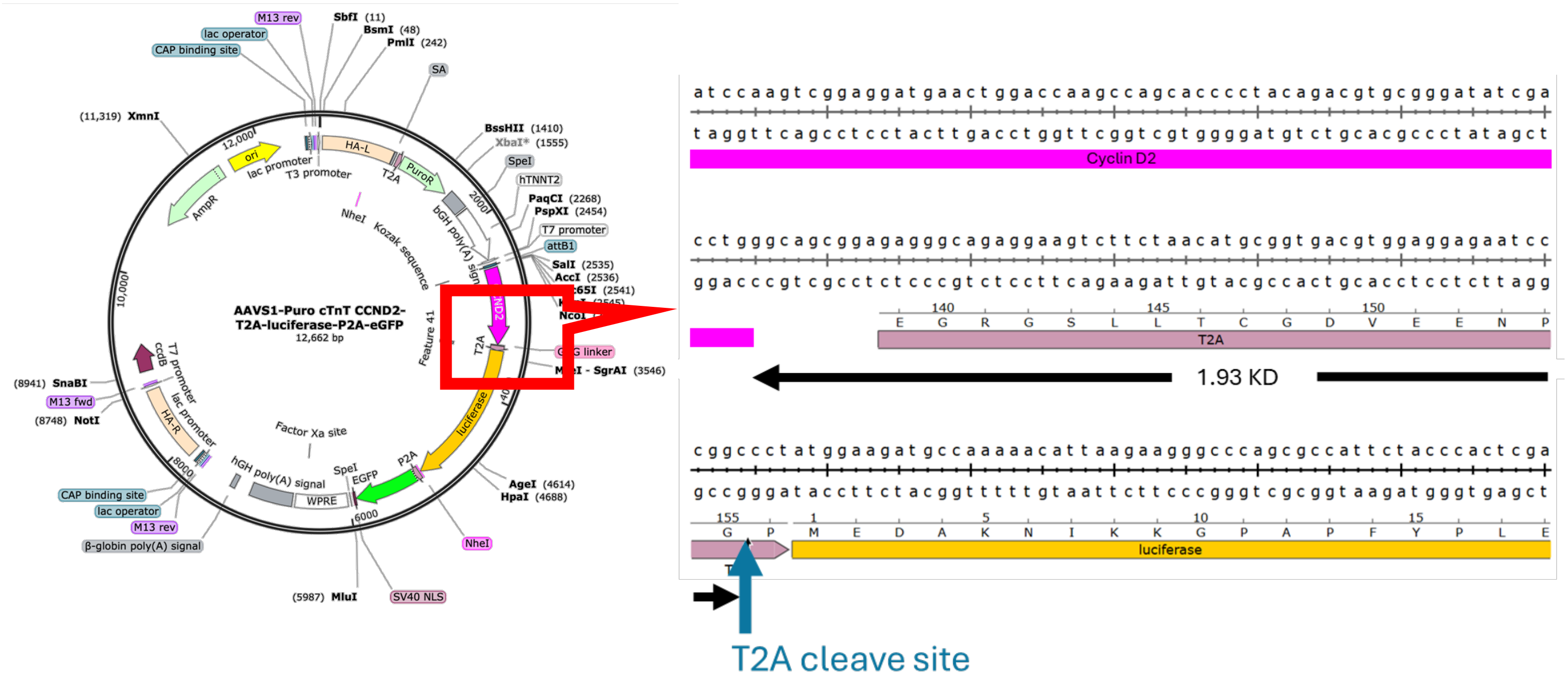

bioRxiv preprint doi: https://doi.org/10.1101/2025.04.24.650512; this version posted April 26, 2025. The copyright holder for this preprint (which was not certified by peer review) is the author/funder. This article is a US Government work. It is not subject to copyright under 17 USC 105 and is also made available for use under a CC0 license.

**Supplemental Figure S5.** Induced stable recombinant cyclin D2 expression in canine M5 MM cells (M5D2). A) Alignment of protein sequences of human cyclin D2 (P30279) and canine cyclin D2 (A0A8I3Q7A5) revealed 95% homology. B) Western blot of cyclin D1 and D2 in M5 and M5D2 MM cells with and without the induction cyclin D2 expression. Doxycycline was added to cultures to induce the expression of recombinant cyclin D2 (+) in M5D2 cells. (C) The expression construct included a residual T2A peptide fragment at the carboxy-terminal aspect, resulting in an estimated 1.9 KD greater size molecular weight in human recombinant cyclin D2.

bioRxiv preprint doi: https://doi.org/10.1101/2025.04.24.650512; this version posted April 26, 2025. The copyright holder for this preprint (which was not certified by peer review) is the author/funder. This article is a US Government work. It is not subject to copyright under 17 USC 105 and is also made available for use under a CC0 license.

**Supplemental Table S1. Reagents- siRNA, primers and antibodies**

**siRNA**

| | |
|---|---|
| CCND1 siRNA #1 | CCAAAGUGAUUAAGUGUGAUU |
| CCND1 siRNA #2 | CCGACGAACUGCUGCAAAUUU |
| CCND1 siRNA #3 | UGAACAAACUGAAGUGGAAUU |
| | |
| CCND2 siRNA #1 | GCAGAGAAGUUGUGCAUUUUU |
| CCND2 siRNA #2 | GGUCCAAGUCCGAGGAUGAUU |
| CCND2 siRNA #3 | CCUACAGACGUGCGGGAUAUU |
| CCND2 siRNA #4 | GCUGGAGGUCUGUGAGGAAUU |

**primers**

| | |
|---|---|
| canine CCND1-F1 | CAGTGGCAGAGGAGAACAAA |
| canine CCND1-R1 | GAGGGTGGGTTGGAAATGAA |
| canine CCND2-F1 | CAAGGCTGGGAGAAGAAATAGA |
| canine CCND2-R1 | CTACCACCCATTACCACACTAC |
| canine CCND3-F1 | TACAGGACAGAGCAGGGATAA |
| canine CCND3-R1 | ACCTTGTGGCCTTAGGAAAG |
| canine actin-F1 | CCTGGAGAAGAGCTACGAACTA |
| canine actin-R1 | CCAGGAAAGAAGGTTGGAAGAG |

**Antibodies**

| Target | application | vendor | catalog no. |
|---|---|---|---|
| cyclin D1 | WB, IF | cell signaling | 55506 |
| cyclin D2 | WB | cell signaling | 3741 |
| cyclin D2 | IF | Abcam | ab207604 |
| pERK | WB | cell signaling | 4270 |
| pAKT473 | WB | cell signaling | 4060 |
| actin | WB | Sigma | A5441 |

**Supplemental Table 2. Multiple two-way comparisons between mucosal melanoma cell line viabilities over a range of trametinib concentrations.**

| p values | M1 vs M2 | M1 vs M3 | M1 vs. M5 | M1 vs Jones | M2 vs M3 | M2 vs. M5 | M2 vs. Jones | M3 vs M5 | M3 vs Jones | M5 vs Jones |
|---|---|---|---|---|---|---|---|---|---|---|
| 0.1 μM | 0.881 | 6.75E-04 | 1.79E-12 | 7.06E-12 | 1.75E-03 | 4.06E-08 | 1.18E-07 | 8.32E-11 | 1.09E-09 | 0.955 |
| 0.6 μM | 0.727 | 5.47E-04 | 9.72E-13 | 2.84E-13 | 3.95E-04 | 1.82E-11 | 2.30E-13 | 1.79E-05 | 2.31E-05 | 0.024 |
| 2.5 μM | 0.894 | 3.26E-06 | 7.36E-12 | 1.09E-12 | 5.14E-04 | 6.63E-06 | 5.37E-06 | 1.85E-05 | 1.61E-05 | 0.045 |
| 10.0 μM | 0.752 | 4.34E-06 | 1.26E-18 | 1.32E-17 | 8.50E-06 | 5.77E-06 | 5.14E-06 | 0.036 | 0.095 | 0.169 |

**p values correspond to data display in Figure 1A**

bioRxiv preprint doi: https://doi.org/10.1101/2025.04.24.650512; this version posted April 26, 2025. The copyright holder for this preprint (which was not certified by peer review) is the author/funder. This article is a US Government work. It is not subject to copyright under 17 USC 105 and is also made available for use under a CC0 license.
